# A Novel Small Acid Soluble Protein Variant Is Important for Spore Resistance of Most *Clostridium perfringens* Food Poisoning Isolates

**DOI:** 10.1371/journal.ppat.1000056

**Published:** 2008-05-02

**Authors:** Jihong Li, Bruce A. McClane

**Affiliations:** Department of Microbiology and Molecular Genetics, University of Pittsburgh School of Medicine, Pittsburgh, Pennsylvania, United States of America; The University of Texas-Houston Medical School, United States of America

## Abstract

*Clostridium perfringens* is a major cause of food poisoning (FP) in developed countries. *C. perfringens* isolates usually induce the gastrointestinal symptoms of this FP by producing an enterotoxin that is encoded by a chromosomal (*cpe*) gene. Those typical FP strains also produce spores that are extremely resistant to food preservation approaches such as heating and chemical preservatives. This resistance favors their survival and subsequent germination in improperly cooked, prepared, or stored foods. The current study identified a novel α/β-type small acid soluble protein, now named Ssp4, and showed that sporulating cultures of FP isolates producing resistant spores consistently express a variant Ssp4 with an Asp substitution at residue 36. In contrast, Gly was detected at Ssp4 residue 36 in *C. perfringens* strains producing sensitive spores. Studies with isogenic mutants and complementing strains demonstrated the importance of the Asp 36 Ssp4 variant for the exceptional heat and sodium nitrite resistance of spores made by most FP strains carrying a chromosomal *cpe* gene. Electrophoretic mobility shift assays and DNA binding studies showed that Ssp4 variants with an Asp at residue 36 bind more efficiently and tightly to DNA than do Ssp4 variants with Gly at residue 36. Besides suggesting one possible mechanistic explanation for the highly resistant spore phenotype of most FP strains carrying a chromosomal *cpe* gene, these findings may facilitate eventual development of targeted strategies to increase killing of the resistant spores in foods. They also provide the first indication that SASP variants can be important contributors to intra-species (and perhaps inter-species) variations in bacterial spore resistance phenotypes. Finally, Ssp4 may contribute to spore resistance properties throughout the genus *Clostridium* since *ssp4* genes also exist in the genomes of other clostridial species.

## Author Summary

Spores made by pathogenic *Bacillus* and *Clostridium* spp. contribute to disease transmission. *Clostridium perfringens* food poisoning (FP) isolates typically produce spores with exceptional resistance to heat and sodium nitrite. This spore resistance probably facilitates FP strain survival in improperly cooked/held foods, contributing to *C. perfringens* FP outbreaks, which rank among the most common food-borne diseases in developed countries. Currently, the mechanistic basis of the resistant spore phenotype of FP strains is unknown. Here, we report the identification of a novel small acid soluble protein, named Ssp4, and show that sporulating cultures of FP strains producing resistant spores express an Ssp4 variant with Asp at residue 36, while sporulating cultures of *C. perfringens* strains producing sensitive spores express an Ssp4 with Gly at residue 36. We now demonstrate that i) the Ssp4 Asp variant is required for extreme spore resistance of FP strains and ii) this protein may help protect FP strains via tighter DNA binding than the Ssp4 Gly variant. Our study provides important insights into the transmission of a common FP agent and may suggest strategies to interfere with resistant spores of FP strains. These findings may also have relevance for other pathogenic *Clostridium* spp. carrying an *ssp4* gene.

## Introduction

*Clostridium perfringens* is the 2^nd^ most commonly-identified agent of bacterial food poisoning (FP) in the USA and UK, where (respectively) 250,000 or 85,000 cases of *C. perfringens* FP occur annually [1],[2],[3]. *C. perfringens* FP also currently ranks as the second or third leading cause of food-borne death in (respectively) the UK or USA [2],[3], mainly in the elderly or debilitated. Economic losses (medical care and lost productivity) from this single FP amount to several hundred million dollars per year [1].

The gastrointestinal symptoms of *C. perfringens* FP are caused by *C. perfringens* enterotoxin [4]. The enterotoxin gene (*cpe*) can be either chromosomal or plasmid-borne, but most FP isolates carry only a chromosomal *cpe* gene [5]–[8]. Those typical FP strains with a chromosomal *cpe* gene also produce spores that are extremely resistant to such common food hygiene approaches as cooking, holding foods at elevated or low temperatures, and the addition of chemical preservatives to foods [9]–[11]. For example, the spores of FP strains carrying a chromosomal *cpe* gene exhibit, on average, 60-fold higher decimal reduction values at 100°C (D_100_ value, i.e., the time a culture must be held at 100°C to reduce viability by 90%) than either the spores of isolates carrying a plasmid-borne *cpe* gene or spores of *cpe-*negative *C. perfringens* isolates [9]. This exceptional spore resistance is thought to favor the survival of typical FP strains in improperly cooked, prepared, or stored foods, which represent the most common transmission vehicles for *C. perfringens* FP.

No explanation has yet been offered for the resistance phenotype of spores made by typical FP strains. While α/β-type small acid soluble proteins (SASPs) have been associated with spore heat and nitrite resistance in both *C. perfringens* and *Bacillus* spp. [12], a previous study reported that the three known *C. perfringens* α/β-type SASP genes (*ssp1*, *ssp2* and *ssp3*) share identical sequences. Furthermore, these three *ssp* genes are expressed at similar levels in several *C. perfringens* isolates, including F4969 and SM101, which (respectively) produce sensitive or resistant spores [13],[14]. Therefore, the current study utilized the recently-sequenced genome of *C. perfringens* strain SM101 [15] to identify an additional ORF with homology to a novel α/β-type SASP. We now present evidence that variants of this novel *C. perfringens* SASP, which we are naming Ssp4, are important for the resistant spore phenotype exhibited by most *C. perfringens* FP strains carrying a chromosomal *cpe* gene.

## Results

### Identification of a Novel, Putative SASP-Encoding ORF in *C. perfringens* Isolates

Since previous studies [13],[14] had reported that the ORF sequences of the three known *C. perfringens* SASP-encoding genes are identical in several *C. perfringens* isolates (including SM101 and F4969), the current study first confirmed those prior findings by extending *ssp1, ssp2*, and *ssp3* sequencing analyses to several additional *C. perfringens* isolates that had previously been well-characterized for their production of resistant or sensitive spores [9]–[11],[13],[14]. No sequence differences were detected in the three known *ssp* ORFs among FP strains 191-10, NCTC8239, NCTC10239 and SM101, which each carry a chromosomal *cpe* gene and produce resistant spores, and nonfoodpoisoning (NFP) isolates NB16, T34058, F4969, F5603, 222, ATCC3624 and ATCC13124, which each produce sensitive spores.

**Figure 1:**
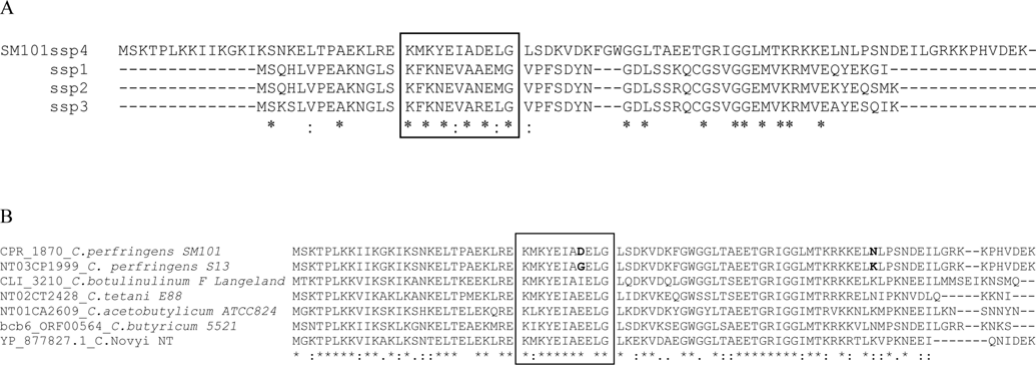
Ssp4 alignment versus other *C. perfringens* SASPs (Panel A) or Ssp4 homologues in other *Clostridium* spp. (Panel B). Box shows the conserved region common to all SASPs. Bold residues represent the variant residues in Ssp4 of F4969 and SM101. Sequences were obtained from [12],[16],[19].

Therefore, a bioinformatic search [16] was performed on the recently-completed genome sequence of *C. perfringens* strain SM101 [15], which is a transformable derivative of a FP strain and produces resistant spores. This search identified an SM101 ORF (CPR_1870) that, at the initiation of this work, was annotated as a ribosomal protein but has since been re-annotated as possibly encoding a novel α/β-type SASP. This putative SASP-encoding ORF, which is being named *ssp4*, is clearly distinct from the recognized ORFs encoding the three previously-identified SASP proteins of *C. perfringens*. The ORF is predicted to encode a 90 amino acid protein of ∼10.2 kDa sharing only 18.9%, 20.9% and 20.9% identity, respectively, with the ∼6.7 kDa Ssp1, Ssp2 and Ssp3 proteins that are each comprised of 59 or 60 amino acids (Fig. 1A).

The sequence of this novel *ssp4* ORF was found to be identical in all eleven initially-surveyed *C*. *perfringens* isolates, except for differences at two codons. Those sequence variations resulted in two different amino acids being consistently present at Ssp4 residue 36 and 72 between the four initially-surveyed FP isolates versus the seven surveyed NFP isolates (Table 1).

**Table 1:** Isolates used in this study.

Isolate	Genotype	Heat resistance^1^	SASP specify sequence		Source
			36 residue	72 residue	
NB16	Plasmid *cpe*, IS*1151*	-	Gly	Lys	NFP [9]
T34058	Plasmid *cpe*, IS*1470-like*	-	Gly	Lys	NFP [9]
F4969	Plasmid *cpe*, IS*1470-like*	-	Gly	Lys	NFP [9]
F5603	Plasmid *cpe*, IS*1151*	-	Gly	Lys	NFP [9]
222	Plasmid *cpe*, IS*1470-like*	-	Gly	Lys	NFP [9]
01E803	Chromosomal *cpe*	-	Gly	Lys	FP [18]
01E809	Chromosomal *cpe*	+	Asp	Lys	FP [18]
191-10	Chromosomal *cpe*	+	Asp	Asn	FP [9]
NCTC8239	Chromosomal *cpe*	+	Asp	Asn	FP [9]
NCTC10239	Chromosomal *cpe*	+	Asp	Asn	FP [9]
SM101	Chromosomal *cpe*	+	Asp	Asn	FP [15]
ATCC3264	*cpe* negative	-	Gly	Lys	NFP [9]
ATCC13124	*cpe* negative	-	Gly	Lys	NFP [9]

All isolates classify as type A (data not shown).

1 This study and [9].

### Expression of the *ssp4* Gene by *C. perfringens* Isolates SM101 and F4969

**Figure 2:**
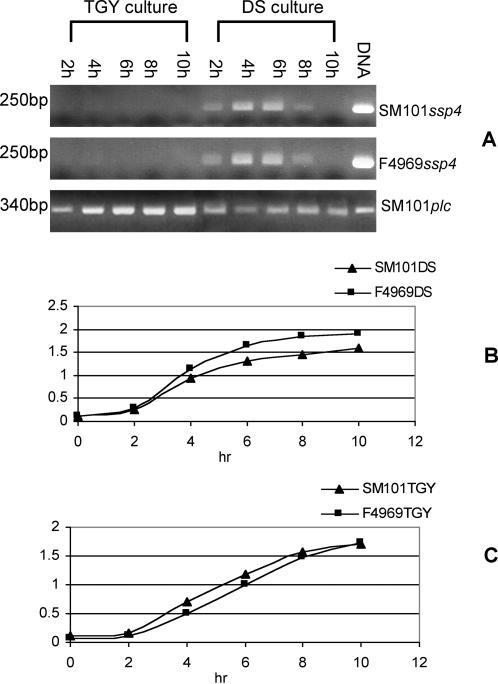
Expression of the *ssp4* gene by wild-type F4969 or SM101 during vegetative growth and sporulation. Panel A, RT-PCR analyses of SM101 *ssp4*, SM101 *plc*, or F4969 *ssp4* expression using cultures grown for 2–10 h in TGY (for vegetative growth) or Duncan-Strong sporulation medium (DS). Panel B and C show post-inculation change in optical density (OD_600_) for cultures of SM101 or F4969 growing in, respectively, TGY or DS medium.

The apparent correlation shown in Table 1 between *ssp4* ORF sequence differences and spore sensitivity or resistance suggested that the newly-identified, putative *ssp4* ORF might encode a key protein contributor to the resistant spore phenotype of typical FP strains. To test this hypothesis, we first evaluated by RT-PCR (Fig. 2) whether the *ssp4* ORF is expressed by two transformable *C. perfringens* isolates, i.e., SM101 and F4969, that (respectively) are known to produce resistant or sensitive spores ([17] and Table 2). Since expression of the *ssp1*, *ssp2* and *ssp3* genes of *C. perfringens* is reportedly sporulation-associated [13], this RT-PCR study also analyzed whether *ssp4* expression, if any, occurs in exponentially growing vegetative cultures or sporulating cultures of SM101 and F4969.

**Table 2:** Spore resistance to heat (100°C) and sodium nitrite (nitrous acid).

Strain	Description	Heat shock (100°C)	Nitrous acid treatment
		D value (min)	Log reduction after 60 min
F4969 WT	Wild type	0.5±0.0	4.0±0.5
F4969::*ssp4*	F4969 *ssp4* null mutant	0.5±0.0	5.1±0.4
F4969::*ssp4*(pCS)	F4969::*ssp4* + pJIR751CS 1	14.0±1.4	3.1±0.2
F4969::*ssp4*(pCF)	F4969::*ssp4* + pJIR751CF 1	0.5±0.0	3.8±0.3
F4969::*ssp4*(pJIR751)	F4969::*ssp4* + pJIR751	0.5±0.0	4.9±0.5
F4969::*ssp4*(pCO)	F4969::*ssp4* + pJIR751CO 1	10.0±2.0	3.4±0.4
SM101 WT	Wild type	59.1±1.3	1.1±0.4
SM101::*ssp4*	SM101 *ssp4* null mutant	8.7±1.9	4.0±0.1
SM101::*ssp4*(pCS)	SM101::*ssp4* + pJIR751CS	44.7±1.8	1.1±0.6
SM101::*ssp4*(pCF)	SM101::*ssp4* + pJIR751CF	16.4±0.6	3.2±0.1
SM101::*ssp4*(pJIR751)	SM101::*ssp4* + pJIR751	9.3±1.1	3.8±0.0
SM101::*ssp4*(pCO)	SM101::*ssp4* + pJIR751CO	38.0±1.7	2.2±0.3
01E809	Wild type	50.0±3.3	1.5±0.5
01E803	Wild type	0.7±0.2	3.7±0.2

1 pCS, pCF and pCO are the shuttle plasmid pJIR751 carrying the cloned *ssp4* gene (upstream sequence and ORF) from, respectively, SM101, F4969 or 01E809.

Results from these RT-PCR studies (Fig. 2) clearly demonstrated that *ssp4* expression becomes detectable within 2 h after inoculation of either SM101 or F4969 into Duncan-Strong (DS) sporulation medium. Expression of the *ssp4* gene then peaked between 4–6 hours post-inoculation in both SM101 and F4969 DS cultures. For comparison, the first visible forespores and phase-refractile spores of SM101 or F4969 appeared, respectively, within ∼6–8 h using these culture conditions.

However, RT-PCR detected only weak (if any) *ssp4* expression by vegetative cultures of either SM101 or F4969 growing in TGY medium (Fig. 2). When detected, this limited *ssp4* expression in TGY cultures peaked during the log phase of exponential growth, i.e., at ∼4 h post-inoculation, for both strains. In contrast to the poor (if any) *ssp4* expression observed using RNA isolated from TGY cultures of F4969 or SM101, RT-PCR detected strong expression of the *plc* gene using those same TGY RNA preparations, confirming that those RNA preparations were valid for detecting gene expression by TGY cultures. For completeness, *plc* expression was also demonstrated using RNA isolated from DS cultures of these two isolates (Fig. 2 and data not shown).

Consistent with the RT-PCR results of Fig. 2, Western blot analysis demonstrated substantial Ssp4 production by DS cultures of both SM101 and F4969, but detected only trace amounts (if any) of Ssp4 production in TGY cultures of those isolates (data not shown). No forespores or spores were visible in either the SM101 or F4969 TGY cultures and no colonies grew after heat-shocking (70°C for 20 min) of aliquots from these exponentially growing vegetative cultures.

### Inactivation of the *ssp4* Gene and Phenotyping of Those *ssp4* Null Mutants

**Figure 3:**
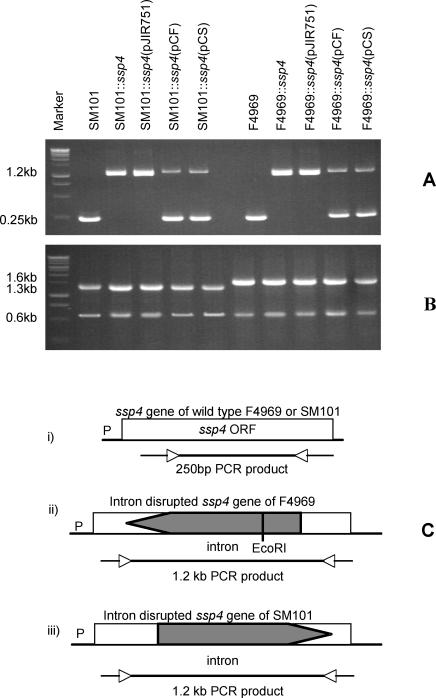
Intron-based mutagenesis to create SM101 and F4969 *ssp4* null mutants. Panel A, *ssp4*-specific PCR results for: wild-type SM101; SM101::*ssp4*, the SM101 *ssp4* null mutant; or SM101::*ssp4* transformed with pJIR751, pJIR751 carrying the SM101 *ssp4* gene, or pJIR751 carrying the F4969 *ssp4* gene; a blank lane; wild-type F4969; F4969::*ssp4*, the F4969 *ssp4* null mutant; F4969::*ssp4* transformed with the pJIR751 shuttle plasmid, pJIR751 carrying the F4969 *ssp4* gene, or pJIR751 carrying the SM101 *ssp4* gene. Presence of the larger (∼1.2 kb) PCR product reflects intron disrupted *ssp4* gene, as depicted in Panel C. Panel B shows *cpe* genotyping PCR [8] results confirming that all F4969 or SM101 derivatives still carry, respectively, a plasmid *cpe* gene or a chromosomal *cpe* gene. The left-pointing arrow for F4969 depicts an antisense intron insertion while the right-pointing arrow for SM101 depicts a sense intron insertion. Bars underneath i, ii, and iii of Panel C indicate expected PCR product sizes using B1F and B1R primers.

**Figure 4:**
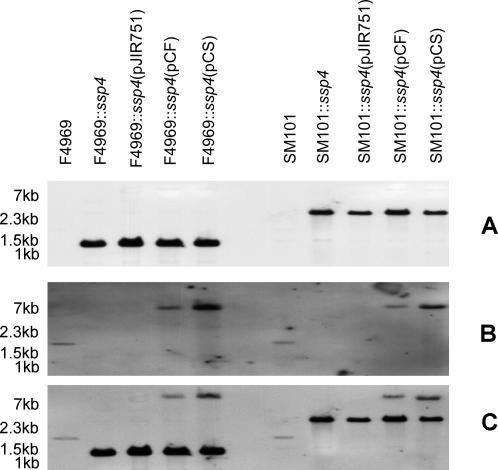
Southern blot analysis of wild-type, *ssp4* null mutants and complementing strains of F4969 or SM101. Panel A shows Southern blot hybridization of a DIG-labeled intron probe. The Southern blot was then stripped and re-probed with a DIG-labeled *ssp4* probe for panel B. Panel C shows an overlay of the A and B blots. DNA size markers are shown at left.

**Figure 5:**
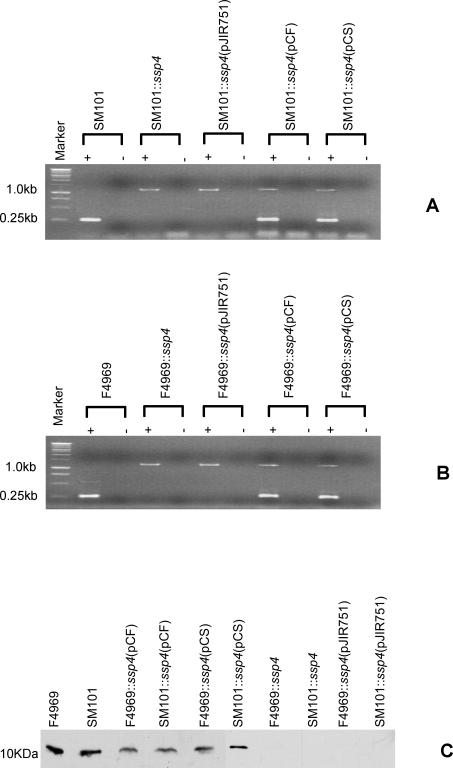
Expression of the *ssp4* gene and Ssp4 production by wild-type, *ssp4* null mutants, and complementing strains of F4969 or SM101. Panel A, RT-PCR analyses for *ssp4* expression by SM101, SM101::*ssp4*, and complementing strains grown for 6 h in DS mediuim. Panel B, RT-PCR analyses of *ssp4* expression by F4969, F4969::*ssp4*, and complementing strains grown for 6 h in DS medium. Lane 1 shows size markers. Lanes labeled “+” were from samples receiving reverse transcriptase, while lanes labeled “-“ lacked reverse transcriptase to show the absence of DNA contamination. Panel C, Western blot analyses for Ssp4 production by overnight DS cultures of wild-type, *ssp4* null mutants or complementing strains of F4969 or SM101.

To directly evaluate whether Ssp4 is important for the resistant phenotype of FP spores, a targeted intron was then used to insertionally-inactivate the *ssp4* gene in both SM101 and F4969. For each resultant mutant, the presence of an intron-inactivated *ssp4* ORF was demonstrated by PCR (Fig. 3), the presence of a single intron insertion in the *ssp4* mutant was shown by Southern blotting (Fig. 4), and the disruption of *ssp4* expression and Ssp4 production by the mutant was proven by RT-PCR and Western blot (Fig. 5).

Phenotypic comparisons then demonstrated that the spores produced by the isogenic SM101 *ssp4* null mutant (SM101::*ssp4*) were considerably less heat- and sodium nitrite-resistant than wild-type SM101 spores (Table 2). These resistance differences are specifically attributable to inactivation of the *ssp4* gene in SM101::*ssp4* since complementing that mutant with the pJIR751 shuttle plasmid carrying the wild-type SM101 *ssp4* gene (creating SM101::*ssp4*(pCS)) substantially restored both spore heat resistance and sodium nitrite resistance. In contrast, only a small increase in spore heat- or sodium nitrite-resistance was detected if the SM101 *ssp4* null mutant was complemented with the same shuttle plasmid carrying the wild-type F4969 *ssp4* gene (creating SM101::*ssp4*(pCF)) and no increased spore resistance to heat or sodium nitrite was observed if SM101::*ssp4* was transformed with the shuttle plasmid alone (creating SM101::*ssp4*(pJIR751). Restored *ssp4* expression and Ssp4 production by all SM101 complementing strains was demonstrated by, respectively, RT-PCR analyses and Western blotting (Fig. 5).

Additionally, complementing a F4969 *ssp4* null mutant (F4969::*ssp4*) with the pJIR751 shuttle plasmid carrying the wild-type SM101 *ssp4* gene (to create F4969::*ssp4*(pCS)) produced spores that were substantially more heat- and sodium nitrite-resistant than the spores made by wild-type F4969 (Table 2). This effect was specific since those F4969::*ssp4*(pCS) spores showed much greater resistance against heat or sodium nitrite than did spores made by F4969::*ssp4* complemented with the wild-type F4969 *ssp4* gene (i.e., F4969::*ssp4*(pCF)), or spores made by the F4969 *ssp4* null mutant transformed with the empty pJIR751 vector (F4969::*ssp4*(pJIR751). Restored *ssp4* expression and Ssp4 production by all F4969 complementing strains was demonstrated by, respectively, RT-PCR analyses and Western blotting (Fig. 5).

The F4969 and SM101 *ssp4* null mutants were both stable over many passages and the complementing plasmids could be packaged inside spores since heat-shocking of complementing strains consistently produced erythromycin-resistant survivors. In addition, all mutants and complementing strains also exhibited similar vegetative growth rates and DS sporulation efficiencies as wild-type SM101 or F4969 (not shown).

### DNA Binding Properties of Ssp4 Variants

**Figure 6:**
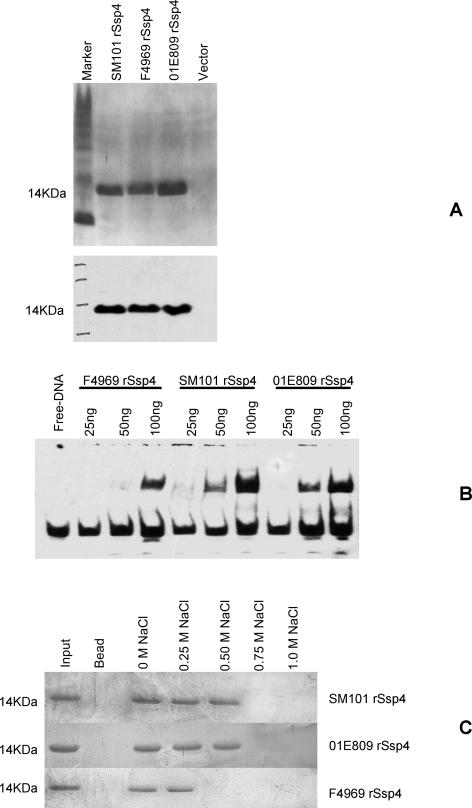
DNA binding properties of purified recombinant His_6_-tagged rSsp4. Panel A, purity and stability of Coomassie blue (top panel) or His_6_-tag Western blotted, purified His_6_-tagged rSsp4 proteins used in Fig. 6 B and C DNA binding experiments. Panel B, EMSA analysis of purified F4969, SM101 and 01E809 rSsp4 binding to *C. perfringens* DNA. Lane 1, free biotin-labeled *C. perfringens* DNA; lanes 2–4, indicated amounts of purified F4969 rSsp4 incubated with *C. perfringens* biotin-labeled DNA; lanes 5–7, indicated amounts of purified SM101 rSsp4 incubated with *C. perfringens* biotin-labeled DNA; and lanes 8–10, indicated amounts of purified 01E809 rSsp4 incubated with *C. perfringens* biotin-labeled DNA. Panel C, effects of NaCl on binding of rSsp4 to DNA. Beads (100 µg) containing calf thymus DNA (Sigma) were incubated with 100 ng of rSsp4 from the indicated strain. After washing with 0, 0.25 M, 0.50 M, 0.75 M or 1.0 M NaCl, the beads were boiled and then analyzed by SDS-PAGE. Lane 1, input protein; lane 2, 100 µg DNA-free beads incubated with 100 ng purified rSsp4 from SM101, 01E809 or F4969; lanes 3–7, 100 µg DNA-containing beads incubated with 100 ng purified rSsp4 from SM101, 01E809 or F4969 and washed with indicated concentrations of NaCl.

Since α/β-type SASPs are thought to protect spores from heat or sodium nitrite by binding to DNA [12], studies were performed to address whether the greater heat and sodium nitrite resistance of spores made by SM101 vs. F4969 might involve stronger DNA binding by the SM101 Ssp4 variant. These DNA binding experiments used highly-purified, recombinant, His_6_-tagged Ssp4 (rSsp4) variants (Fig. 6A).

An electrophoretic mobility shift assay (EMSA) showed the purified SM101 rSsp4 is more effective than the purified F4969 rSsp4 at complexing with, and shifting migration of, *C. perfringens* DNA (Fig. 6B). Also consistent with tighter DNA binding, SM101 rSsp4 remained bound to calf thymus DNA in the presence of NaCl concentrations that caused dissociation of F4969 rSsp4 from the same target DNA (Fig. 6C).

### Analysis of Additional FP Strains Implicates Ssp4 Residue 36 in the Spore Resistance Phenotype of Typical FP Strains

Since the Ssp4s of the seven Table 1 NFP isolates producing sensitive spores and the four initially-studied FP isolates producing resistant spores were identical except for amino acid substitutions at Ssp4 residues 36 (where the four FP strains have an Asp instead of Gly) and 72 (where the four FP strains have an Asn instead of Lys), additional sequencing of the *ssp4* gene (upstream region and ORF) was performed to test whether these same Ssp4 sequence differences hold true for two *C. perfringens* isolates carrying a chromosomal *cpe* gene (data not shown) that had been obtained during a recent Oklahoma food poisoning outbreak [18]. This analysis showed the *ssp4* gene of Oklahoma FP isolate 01E809 is identical to the *ssp4* gene of SM101 except the 01E809 *ssp4* ORF encodes a Lys at Ssp4 residue 72. In contrast, this sequencing revealed that the *ssp4* ORF of Oklahoma FP isolate 01E803 is identical to the *ssp4* ORF of *C. perfringens* isolates producing sensitive spores.

Relative to the two Ssp4 variants made by the initially-studied four FP and seven NFP isolates, the *ssp4* ORF of 01E809 naturally encodes a hybrid Ssp4 variant. Therefore, the spore resistance phenotypes of the two Oklahoma isolates were evaluated, which showed that 01E809 spores are similar in resistance to wild-type SM101 spores and much more resistant than 01E803 spores (Table 2). To directly assess whether the Ssp4 of 01E809 spores can mediate a resistant spore phenotype, the SM101 *ssp4* null mutant was complemented with a shuttle plasmid carrying the 01E809 *ssp4* gene. This complementation yielded spores with a strongly resistant phenotype (Table 2). Similarly, complementation of the F4969 *ssp4* null mutant with the shuttle plasmid encoding 01E809 Ssp4 produced substantially more resistant spores than those of wild-type F4969 or the F4969::*ssp4* mutant complemented with the F4969 *ssp4* gene (Table 2).

The Table 2 data indicated that substitution of an Asp for Gly at Ssp4 residue 36 is important for the resistant spore phenotype among the studied FP strains. Therefore, DNA binding assays were performed to evaluate whether the mechanism of this resistance might involve tighter DNA binding. EMSAs showed that a His_6_-tagged 01E809 rSsp4 variant resembles the SM101 rSsp4 variant (and was more effective than the F4969 rSsp4 variant) with respect to tight binding to *C. perfringens* DNA (Fig. 6B). In addition, calf thymus DNA binding by purified 01E809 rSsp4 and SM101 rSsp4 were similarly NaCl-resistant but those rSsp4s were both more resistant to NaCl-induced dissociation from calf thymus DNA than was F4969 rSsp4 (Fig. 6C).

## Discussion

This study has identified a first explanation for the exceptional spore resistance properties exhibited by most *C. perfringens* FP strains carrying a chromosomal *cpe* gene. We found that a novel SASP protein (now named Ssp4), which is preferentially expressed during sporulation, plays an important role in this spore resistance phenotype. Specifically, strains producing highly resistant spores have an Asp substitution (in place of Gly) at residue 36 of Ssp4. As shown in Fig. 1, residue 36 of Ssp4 is located in a conserved region present in all α/β-type SASPs [12], including Ssp1, Ssp2 and Ssp3 of *C. perfringens*. During spore outgrowth, this conserved SASP region is the site of cleavage by the GPR endoprotease, which exposes DNA for resumption of transcription and provides amino acids for protein synthesis and energy metabolism in the developing vegetative cell [12]. However, participation of this conserved SASP region in DNA protection and spore resistance properties has been less clear [12]. In particular, prior to the current study, the equivalent of Ssp4 residue 36 in α/β-type SASPs had not yet been clearly linked to spore resistance or DNA binding.

Previous studies have shown that variations in SASP levels can impact spore resistance properties. For example, *B. subtilis* spores lacking ∼85% of their α/β type SASPs become more sensitive to DNA-damaging treatments [12]. Furthermore, antisense RNA-induced decreases in levels of the three previously known SASPs produced more heat-sensitive *C. perfringens* SM101 spores [17]. However, to our knowledge, the current findings provide the first indication that natural SASP variants can be important contributors to intra-species variations in spore resistance properties.

Ssp4 may also contribute to spore resistance in other *Clostridium* spp. since bioinformatic searches identified the presence of Ssp4 ORF homologues in other genome-sequenced clostridial species (Fig. 1B). At least five clostridial species, including several major human pathogens and industrially-relevant species, carry an ORF encoding a protein with >70% overall identity to *C. perfringens* Ssp4 [12],[16],[19]. Several additional clostridial species, including the increasingly important pathogen *Clostridium difficile*, carry an ORF encoding a protein with more limited, but still significant, identity to Ssp4. Alignment of Ssp4-like proteins of several clostridial species (Fig. 1B), or even aligning (not shown) all known SASPs made by sporulating bacteria [12], indicated that the presence of an Asp at the equivalent of Ssp4 residue 36 is, thus far, unique to the *C. perfringens* FP strains that carry a chromosomal *cpe* gene and make resistant spores. However, it is notable there is some natural variability at Ssp4 residue 36 among the clostridia (Table. 1). Given this variability, it might be informative for future studies to compare the heat sensitivities of wild-type versus *ssp4* null mutants in other genome-sequenced clostridial strains in order to further elucidate the contribution of Ssp4 (and, possibly, intraspecies Ssp4 variants) to spore phenotypes in other *Clostridium* species.

The current study also revealed that several different amino acids can be present at residue 72 of *C. perfringens* Ssp4. However, those residue 72 Ssp4 variations appear to be less important for resistance properties since both *C. perfringens* sensitive spores and the resistant spores made by strain 01E809 share a Lys at Ssp4 residue 72. The presence of two different amino acids at Ssp4 residues 36 and 72 indicates that *C. perfringens* Ssp4 variants are more common than has been observed, to date, for the highly-conserved Ssp1, Ssp2 and Ssp3 proteins of *C. perfringens* ([14] and this study).

We previously showed [20] that, at the time of retail purchase, ∼1–2% of raw meats, poultry and fish are contaminated with *C. perfringens* isolates carrying a chromosomal *cpe* gene. Every one of those recovered chromosomal *cpe* food isolates formed resistant spores, indicating that spore heat resistance is not selected from a *C. perfringens* population in foods during each cooking or nitrite exposure, but is instead already an intrinsic property of most *C*. *perfringens* isolates carrying a chromosomal *cpe* gene. Coupling that previous finding with the current observation that (despite diverse geographic origins and isolation dates) all of the currently surveyed FP isolates forming resistant spores share a *ssp4* ORF encoding an Asp variant at residue 36 may suggest a common lineage for many typical FP isolates carrying a chromosomal *cpe* gene. Due to competitive advantage in the food poisoning environment from their spore resistance, these typical FP strains forming resistant spores now predominate in the FP environment.

However, our study also provides the first indication that not all wild-type FP isolates carrying a chromosomal *cpe* gene produce resistant spores. This uncoupling of chromosomal *cpe* gene carriage from resistant spore production for isolate 01E803 is consistent with previous results demonstrating that an SM101 *cpe* null mutant still produces highly resistant spores [17]. The presence of different Ssp4 variants in 01E803 and 01E809, two strains that otherwise appear closely-related (if not clonal) and originated from the same FP outbreak involving improperly cooked turkey [18], may reflect a post-cooking reversion of the *ssp4* gene in 01E803 to the Gly Ssp4 variant present in most *C. perfringens*. That revertant may have survived because spore heat resistance was no longer needed after cooking; presumably progeny of 01E803 would be less competitive in future FP events. Since there is no direct linkage between possession of a chromosomal *cpe* gene and formation of a resistant spore, it is possible that selective pressure in the food environment will eventually yield *C. perfringens* FP isolates carrying a plasmidborne *cpe* gene yet producing resistant spores involving Ssp4 variants (a minority of food poisoning cases involve plasmid *cpe* isolates [21],[22]).

Additional studies will be necessary to fully elucidate how Ssp4 variants mediate different *C. perfringens* spore resistance properties, but the detection of DNA binding differences between different Ssp4 variants during the current work suggests one possible mechanism. Furthermore, while the current data clearly demonstrates that Ssp4 variants help to explain isolate-dependent *C. perfringens* spore sensitivity differences, Ssp4 is not the only SASP contributing to *C. perfringens* spore resistance properties. As mentioned earlier, studies from Sarker's group have shown that the three previously known SASPs are also necessary to obtain full resistance for spores made by typical FP strains [14],[23],[24]. Since various SASPs are thought to interact *in vivo* during DNA binding [12], it is possible that the Ssp4 variants identified in this study may display even greater DNA binding differences in the presence of Ssp1, 2 and 3, than was detected by the *in vitro* DNA binding studies of Fig. 6 using only Ssp4. If so, this magnified DNA binding effect might further explain the exceptional resistance properties of some *C. perfringens* spores.

Our determination that the SM101 *ssp4* null mutant still remains substantially more heat-resistant than wild-type F4969, together with previous studies [13],[14],[23] showing similar expression levels of Ssp1, 2 and 3 by SM101 and F4969, may suggest that additional factors beyond the SASPs also contribute to the resistant phenotype of spores produced by many FP strains. Further studies are needed to fully understand all of the mechanisms contributing to the resistant spore phenotype of FP strains, as this knowledge may identify strategies for reducing the incidence of *C. perfringens* type A food poisoning.

## Materials and Methods

### Bacterial strains, media and chemicals

*C. perfringens* type A isolates used in this study are described in Table 1. FTG and TGY broth were used for growing vegetative cultures [9]. Brain heart infusion (BHI) agar was used for plate count analyses [9]. Modified Duncan-Strong (MDS) sporulation medium was used to induce sporulation of *C. perfringens* type A isolates [10]. *E.coli* DH5α was grown at 37°C in LB broth with shaking or on LB agar. Antibiotics were from Fisher Scientific Company.

### Sequencing of the *ssp4* gene in *C. perfringens* isolates

Primers B1F (5′-ATGAGCAAGACACCATTAAAAAA-3′) and B1R (5′-TTACTTTTCGTCA ACGTGAGG-3′) were designed from the *ssp4* gene sequence of *C. perfringens* SM101 (Gene bank accession number CPR_1870) [15]. For each Table 1 isolate, template DNA was obtained from colony lysates [20]. PCR reactions were performed using the following amplification conditions: 94°C for 2 min, 35 cycles of 94°C for 30 sec, 54°C for 30 sec, 72°C for 30 sec, following by a 10 min extension at 72°C. The products were then cloned into pCR®2.1-TOPO vectors (Invitrogen) and sequenced by the University of Pittsburgh Core DNA Sequencing Facility. Unique *ssp4* ORF sequence were deposited in GenBank (accession numbers EU287944 and EU287945).

### RNA extraction and RT-PCR

Wild-type SM101 and F4969 were each grown in TGY for 0–10 h at 37°C. Every 2 h, a 3 ml aliquot of culture was removed and used for RNA extraction with the RiboPure™ Bacteria kit from Ambion, according to the manufacturer's instructions. RNA extractions from mutant or complementing strains used only aliquots from a 6 h TGY culture. RNA with an intron insertion was unstable (data not shown), so only freshly isolated RNA was used for RT-PCR analyses. After 1 h of DNase treatment, RT-PCR reactions were then performed on the RNA samples using the AccessQuick RT-PCR system (Promega). Briefly, 100 ng of each RNA sample were reverse transcribed to cDNA at 45°C for 1 h and then used as template for PCR with primers targeting *ssp4* sequences (as above) or *plc* sequences (cpaF-GCTAATGTTACTGCCGTTGA and cpaR-CCTCTGATACATCGTGTAAG). Control RT-PCR reactions were similarly performed, except for the omission of reverse transcriptase. As an additional control, a PCR amplifying *ssp4* or *plc* sequences was performed using DNA extracted from each strain using the MasterPure Gram-Positive DNA Purification Kit (Epicentre Biotechnologies).

### Construction of *ssp4* null mutants of *C. perfringens* isolates F4969 or SM101

The *ssp4* gene in isolates F4969 or SM101 was insertionally-inactived using a *Clostridium*-modified TargeTron gene knock-out system [25]. Using optimal intron insertion sites identified by the Sigma TargeTron algorithm (www.sigma-genosys.com/targetron/), an intron was targeted to insert, in the antisense orientation, between F4969 *ssp4* ORF nucleotides 47/48 or, in the sense orientation, between SM101 *ssp4* ORF nucleotides 136/137. Primers used for targeting the intron to the F4969 *ssp4* ORF were IBS47 (5′-AAAAAAGCTTATAATTAT CCTTAAATTCCTTATTAGTGCGCCCAGATAGGGTG-3′); EBS47-d (5′-CAGATTGTACA AATGTGGTGATAACAGATAAGTCTTATTAGATAACTTACCTTTCTTTGT-3′); and EBS47 (5′-TGAACGCAAGTTTCTAATTTCGGTTGAATTCCGATAGAGGAAAGTGTCT-3′) or SM101 *ssp4* ORF are IBS136 (5′-AAAAAAGCTTATAATTATCCTTAATAAGATTGA TAGTGCGCCCAGATAGGGTG-3′); EBS136-d (5′-CAGATTGTACAAATGTGGTGATAAC AGATAAGTCTTGATAAATAACTTACCTTTCTTTGT-3′); EBS136 (5′-TGAACGCAAGTT TCTAATTTCGATTCTTATTCGATAGAGGAAAGTGTCT-3′). The 350-bp PCR products were inserted into pJIR750ai [25].

The resultant plasmids, named pJIR750*ssp4*anti and pJIR750*ssp4*sense, were electroporated, respectively, into F4969 or SM101. The transformation efficiency for SM101 was 1.5×10^−6^ or 5×10^4^ transformants/µg plasmid DNA. For F4969, the transformation efficiency was 4×10^−5^ or 1×10^6^ transformants/µg DNA. Transformants selected on BHI agar plates containing 15 µg/ml of chloramphenicol were PCR-screened for an intron-disrupted *ssp4* gene using primers B1F and B1R. A digoxigenin-labeled *ssp4* probe was prepared using primers KO-IBS and KO-EBS1d [26]; that probe was employed for Southern blotting to confirm the presence of a single intron insertion in SM101::*ssp4* and F4969::*ssp4*.

### Construction of *ssp4* complementing strains

The *ssp4* gene (ORF and ∼250 bp of upstream region) was PCR-amplified from SM101, F4969 or 01E809 using primers Spro-F (5′- CCACGAATTCAATATCCCTCCTAAATATAATC-3′) and Spro-R (5′- TAGAGGATCCTTAAATCCCCCATATATTATTC-3′). After digestion with EcoRI and BamHI, those products were separately cloned into the shuttle plasmid pJIR751, creating pCS, pCF or pCO. The F4969 and SM101 *ssp4* null mutants were then individually transformed by electroporation with pJIR751, pCS, pCF or pCO and transformants were selected on BHI agar plates containing 30 µg/ml of erythromycin.

### Expression of recombinant, His_6_-tagged Ssp4 (rSsp4) by *E. coli*

The *ssp4* ORFs of SM101, F4969 or 01E809 were separately cloned into the *E. coli* expression vector pTrcHis A (Invitrogen) using primers SASPC-F (5′- CATGGGATCCATGAGCAAGA CACCATTAAA-3′) and SASPC-R (5′- CATCAAGCTTTTACTTTTCGTCAACGTGAGG). The resultant plasmids were then transformed into *E. coli* DH5α. Those transformants were grown at 37°C to an OD_600_ of 0.6 and then induced with 1 mM isopropyl-β-D-thiogalactopyranoside (Sigma Aldrich), followed by continued growth at 37°C for an additional 4 h. rSsp4 was purified from lysates of the induced transformants with a Ni-NTA spin kit (Qiagen) using a modified native elution buffer (50 mM NaH_2_PO_4_, 30 mM NaCl and 250 mM imidazole, pH 8.0). rSsp4 purity was assessed by Coomassie blue R250 staining samples run on an SDS-PAGE gel and degradation was analyzed by Western blot analysis using a mouse monoclonal antibody against polyHistidine (Sigma Aldrich).

### Western blot analysis of Ssp4 protein Production and Presence in Spores

To produce Ssp4 antibodies, Rabbits were immunized with the highly-purified SM101 rSsp4 shown in Fig. 6A. This 28 day rapid immunization was performed by Pocono Rabbit Farm and Laboratory (Canadensis, PA), an AAALAC-approved, USDA-licensed and OLAW-assured facility.

Unless otherwise specified, Western blot analyses using the Ssp4 antiserum involved inoculating a 0.2 ml aliquot of an FTG culture of a wild-type parent, null mutant or complementing strain into 10 ml of DS medium. After overnight incubation at 37°C, the DS cultures were examined by phase-contrast microscopy to verify sporulation had occurred. The culture was then centrifuged and the pellet washed twice with PBS. The pellet was then resuspended in 0.5 ml of SDS sample buffer and boiled for 10 min. The boiled samples were then microfuged and 20 µl of the supernatant was subjected to Western blotting, as previously described [27].

### rSsp4 DNA binding assays

A previously described protein: DNA binding assay [28] was modified by incubating purified His_6_-tagged rSsp4 (100 ng) from F4969, SM101 or 01E809 for 1 h at 37°C with 100 µg of either empty cellulose beads or cellulose beads containing bound double-stranded calf thymus DNA (Sigma) in binding buffer containing 10 mM Tris-maleate (pH6.7), 50 mM potassium acetate, and 10% glycerol. The beads were then washed three times with binding buffer before sequential washes with 0.25 M, 0.50 M, 0.75 M, and 1.0 M NaCl. After each NaCl wash, an aliquot of beads was removed and resuspended in SDS-PAGE sample buffer, boiled for 5 min; after centrifugation, the supernatant was analyzed by SDS-PAGE, followed by silver staining.

### Electromobility shift assays (EMSAs)

A 3′- biotin-labeled probe consisting of a 55 bp sequence of the *cpe* gene was prepared using primers Label-D (5′-TTAGGAAATATTGATCAAGGTTCATTAATTGAAACTGGTGAAAG ATGTGTTTTAA-3′) and Label-R (5′-TTAAAACACATCTTTCACCAGTTTCAATTAATGA ACCTTGATCAATATTTCCTAA-3′) and a biotin 3′-end DNA labeling kit (Pierce). This probe was then used in a modified version of a previously-described EMSA [29], which involved incubating 1 µl of probe with 25, 50 or 100 ng of purified SM101, F4969, or 01E809 His_6_-tagged rSsp4 protein at 37°C for 1 h, then fixing any rSsp4 bound to DNA by the addition of glutaraldehyde (final concentration of 0.01% (v/v)) for 15 min incubation at 37°C. Those mixtures were loaded onto a 6% polyacrylamide gel and electrophoresed in 0.5× TBE (Tris-borate-EDTA) buffer at 4°C for 1 h. DNA-protein complexes were transferred to a positive charge nylon membrane (Roche Applied Science) and detected with a LightShift Chemiluminescent EMSA kit (Pierce).

### Measurement of spore resistance to heat and sodium nitrite

The resistance of *C. perfringens* spores to moist heat was determined as described previously [9]. To evaluate sodium nitrite (nitrous acid) resistance, we modified a previous assay [30] by incubating a 1 ml aliquot of pelleted spores in 100 µl of 100 mmol NaNO_2_, 100 mmol Na acetate (pH4.5) at room temperature for 60 min; aliquots were then diluted 10 fold in 500 mmol KPO_4_ (pH8.5). After mixing and centrifugation, the pellet was washed with 1 ml of sterile water and then resuspended in 1 ml of water. The spore suspension was heated at 75°C for 20 min to kill the remaining vegetative cells. These suspensions were then serially diluted from 10^−2^ to 10^−7^ with sterile water and plated on BHI agar plates, which were incubated anaerobically overnight at 37°C prior to colony counting.

### Growth rate measurements for wild type, mutant and complementing strain

Vegetative growth of wild-type, mutant and complementary strains was determined as described previously [10].
